# Annealing effect on the structural and optical behavior of ZnO:Eu^3+^ thin film grown using RF magnetron sputtering technique and application to dye sensitized solar cells

**DOI:** 10.1038/s41598-020-65231-6

**Published:** 2020-05-22

**Authors:** Francis Otieno, Mildred Airo, Rudolph M. Erasmus, Alexander Quandt, David G. Billing, Daniel Wamwangi

**Affiliations:** 1 Molecular Science Institute, School of Chemistry, University of the Witwatersrand, Private Bag 3, Wits, 2050 Johannesburg, South Africa; 2 Material Physics Research Institute (MPRI), School of Physics, University of the Witwatersrand, Private Bag 3, Wits, 2050 Johannesburg, South Africa

**Keywords:** Materials science, Nanoscience and technology, Physics

## Abstract

Eu-doped ZnO (ZnO:Eu^3+^) thin films deposited by RF magnetron sputtering have been investigated to establish the effect of annealing on the red photoluminescence. PL spectra analysis reveal a correlation between the characteristics of the red photoluminescence and the annealing temperature, suggesting efficient energy transfer from the ZnO host to the Eu^3+^ ions as enhanced by the intrinsic defects levels. Five peaks corresponding to ^5^D0–7F_J_ transitions were observed and attributed to Eu^3+^ occupancy in the lattice sites of ZnO thin films. As a proof of concept a dye sensitized solar cell with ZnO:Eu^3+^ thin films of high optical transparency was fabricated and tested yielding a PCE of 1.33% compared to 1.19% obtained from dye sensitized solar cells (DSSC) with pristine ZnO without Eu produced indicating 11.1% efficiency enhancement which could be attributed to spectral conversion by the ZnO:Eu^3+^.

## Introduction

Zinc oxide has emerged as a crucial member of the group II–VI family owing to its attractive properties such as a wide band gap (3.37 eV) and a large excitonic binding energy (60 meV)^1–4^ as well as being environmental friendly material^5^. Highly transparent and conductive ZnO thin film with nanostructured morphologies is an extensively researched field for potential technological applications, especially for the use in various electronic and optoelectronic devices, such as surface acoustic wave devices, gas sensing applications, transparent coating and solar cell applications^6^. Various growth techniques have been reported including chemical vapor deposition (CVD), thermal oxidation, pulsed laser deposition, spray pyrolysis and sputtering^7^. Among these methods, magnetron sputtering of transparent conductive oxides remains a promising technique capable of low temperatures deposition of thin films with good optical and electronic properties with a special advantage of the scalability to large areas^8^.

The use of high quality II–VI semiconductor nanocrystals as dopant hosts for optically active impurities acting as luminescence centers has gained a lot of interest due to efficient luminescence even at room temperature^9,10^. Such dopants are rare-earth ions which are optically and magnetically active in the semiconductor (ZnO) host crystals^11^ such that, strong interactions between the quantum-confined carriers and localized electrons on impurities from impurity-doped ZnO will produce efficient photoluminescence. This is because direct excitation of the parity forbidden 4f–4 f transitions of Eu^3+^ is generally inefficient compared with the host absorption in UV region^12^. Among the rare earth ions, Eu has emerged as an attractive dopant for red emission in the range of 620–740 nm and the resulting related luminescence lines have been reported to be strongly dependent on the structural quality and thermal cycling of the host material^13^. Upon resonance excitation hot carriers-impact excitation, or carriers injected in the p-n junction structure, ZnO semiconductor has the potential to display luminescence over wavelengths from UV to infrared with potential applications in electroluminescence (EL) and cathodoluminescence (CL) displays^14^.

In the present work, we report on the structural, morphological and optical changes on the preparation of transparent conducting thin of ZnO:Eu^3+^ thin film. Thus, establish the dependence of the enhancement of 5D0 → 7F2 transition in PL spectrum on annealing temperature. In this study, Eu doped ZnO thin films are grown using RF magnetron sputtering at room temperature. The films were subsequently studied after annealing in argon in the range 500–900 °C. Moreover, the blue and green emissions in ZnO:Eu^3+^ nanostructures related to native ZnO defect are also discussed.

## Experimental procedure

### Thin film growth

ZnO (97%): Eu (3%) (99.99% purity) target of diameter 76 mm and thickness 6 mm sourced from Semiconductor Wafer, Inc. (SWI) have been used for thin film deposition. This was carried out using R.F. magnetron sputtering system. The substrate used were p-type (001) Si, quartz (for optical measurements) and FTO coated glass (for device fabrication). The substrates were thoroughly cleaned with organic solvents and dried before loading in the sputtering system. The vacuum chamber was evacuated using a rotary roughing pump to a base pressure of 2 × 10^−2^ mbar before further pump down by turbomolecular pump to 1.8 × 10^−5^ mbar. Magnetron sputtering was carried out in an argon gas atmosphere supplied into the chamber through a constant precision leak valve at a flow rate of 13.0 sccm.

### Characterization techniques

The surface morphology of the films were characterized using FEI Nova Nanolab 600 SEM. Rutherford backscattering spectrometry (RBS) measurements were performed to determine the thickness and distribution profiles of Eu^3+^ ions in the ZnO matrix annealed 500 °C using 4He^+^ particles of energy 1.6 MeV at a backscattering angle of 165° (IBM geometry). The crystallographic properties of the ZnO films was carried out using GXRD Bruker D8 Discover, 40 kV, 40 mA using a 8.0 keV Cu-Kα radiation. The scanning range of 2θ was varied from 10° to 80° at a low scanning rate of 1.2°/minute. Photoluminescence measurements were carried out using a Horiba LabRAM HR spectrometer with a 150 lines/mm grating and an excitation wavelength of 244 nm from a frequency doubled Lexel argon ion laser

### Solar cell fabrication

As a proof of concept of transparent conducting and spectral conversion layer, a dye sensitized solar cell was fabricated using FTO electrode annealed at 500 °C chosen to avoid glass softening at higher temperatures. For sensitization, the ZnO and ZnO:Eu^3+^ films were quickly thoroughly deeply dipped into a dye solution (3.0 × 10^−4^ M mixture of the ruthenizer 535-bisTBA (N-719) in methanol) before drying for 12 h at room temperature. The counter electrode was composed of a thin layer of Pt (50 nm) sputter coated on a clean FTO substrate.

**Scheme 1:**
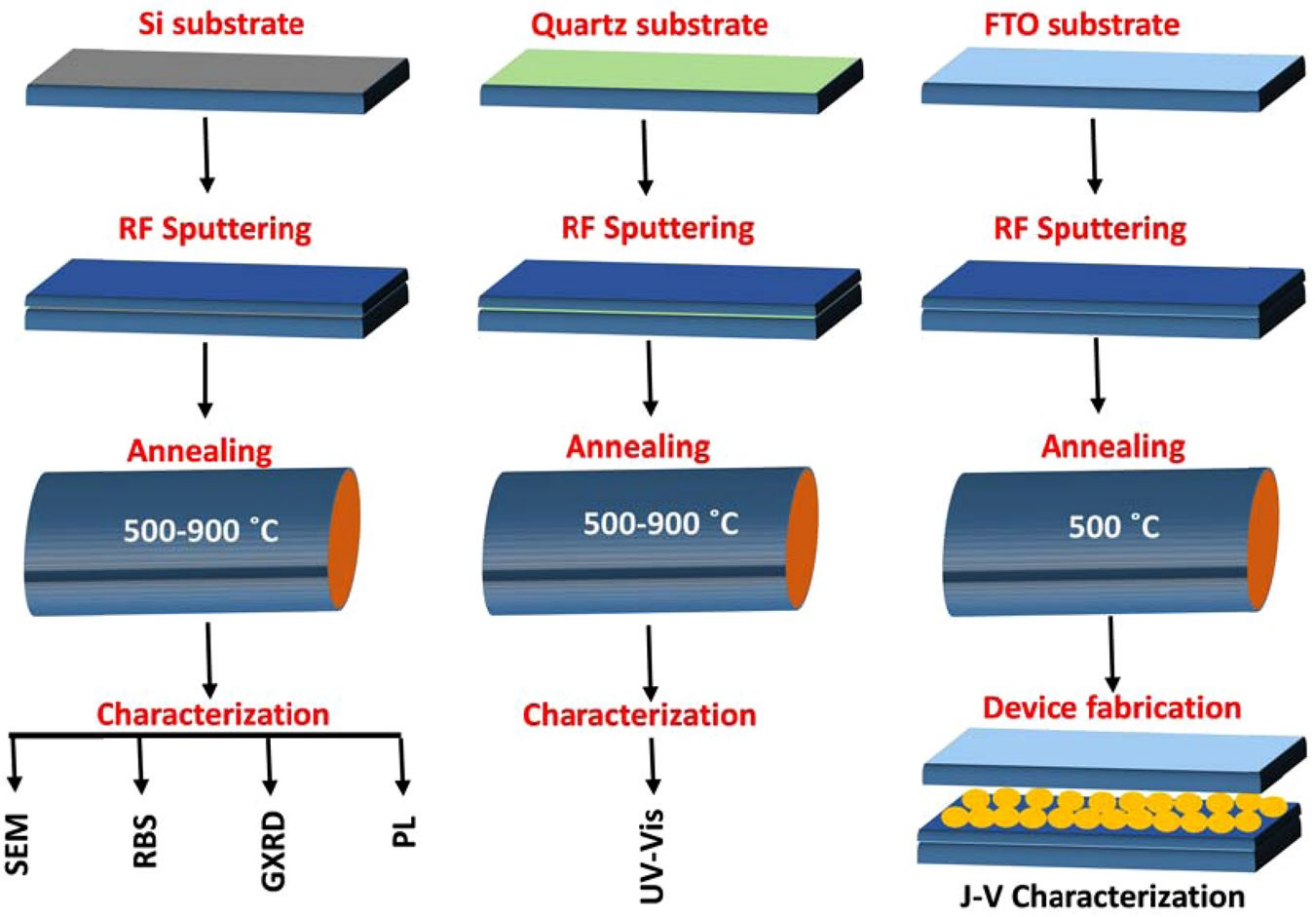
Schematic description of the procedure for thin films making characterization and device.

The electrode with ZnO and ZnO:Eu^3+^ thin film photoanode was placed face up on a flat surface, and the counter electrode placed on top of it. The two opposing glass plates were then offset from one another to enable complete coverage of photoanode by the counter electrode. The redox electrolyte solution (I^−^/I_3_^−^), made of 0.6 M methylhexylimidazolium iodide, 0.1 M iodine, 0.5 M tert-butylpyridine, and 0.1 M lithium iodide in 3-methoxypropionitrile, was then placed on the edges of the plates before the liquid was drawn into the space between the electrodes via capillary action. An epoxy adhesive was utilized to hold/seal the electrodes together. The entire thin film making and characterization is summarized in Scheme 1.

## Results and discussion

### Morphological studies

**Figure 1:**
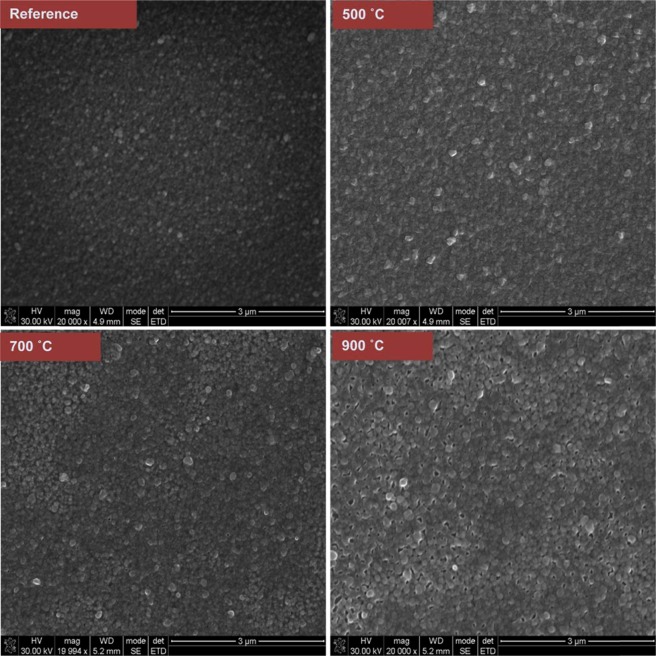
Scanning Electron Microscopy micrographs of Eu doped ZnO films annealed at temperature range of 500–900 °C.

Figure 1 shows the SEM image of ZnO:Eu^3+^ annealed at different temperatures ranging between 500–900 °C.

The micrographs of both pristine and annealed thin films shown in Fig. 1 indicate a uniform deposition of compact columnar growth of ZnO:Eu^3+^ thin films. The films have fine grain structure with average grain size, calculated from Scherrer’s formula, in the range of 51.5 to 70.4 nm. The increase in temperature leads to an enhancement in the grain size and coverage. This is coupled with an increase in surface roughness as witnessed using tapping mode AFM (see Supplementary Fig. S1). This change may be attributed to the stress in the thin films prompting a disturbance of the columnar growth.

### Rutherford Backscattering Spectroscopy Measurements

**Figure 2:**
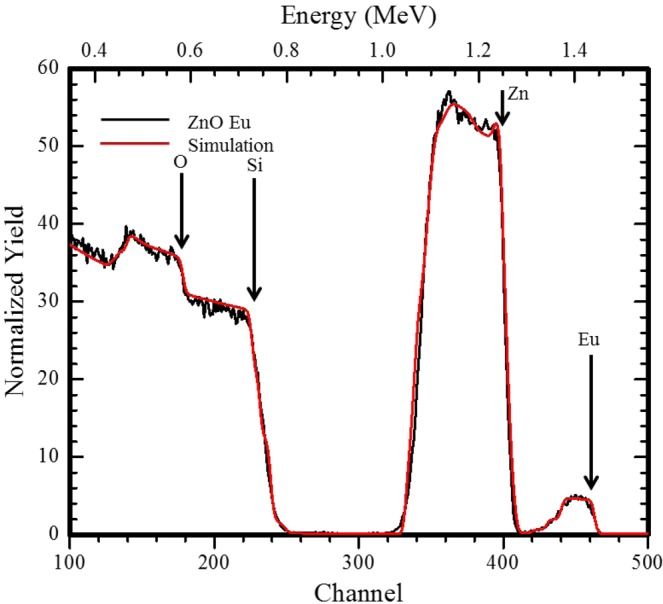
Random and simulated RBS spectra from the ZnO:Eu^3+^ thin film annealed at 500 °C.

The elemental and percentage composition and distribution of the Eu^3+^ ions in the ZnO thin film was established using Rutherford Backscattering Spectroscopy as shown in Fig. 2 for film annealed at 500 °C. The elements could also be confirmed by EDS (see Supplementary Fig. S2).

Figure 2 shows the raw (black curve) and simulated (red curve) RBS spectra of ZnO:Eu^3+^ thin film annealed at 500 °C. The vertical arrows indicate the scattering energies of the elemental constituents in the film. The simulation of the experimental spectra has been carried using the XRump code^15^ and the results of their depth profile are presented in Table 1.

**Table 1:** Derived atomic composition at various film depth from XRump code.

layer	Depth (nm)	Sublayers composition			
		Zn	O	Eu	Si
1	118.00	0.475	0.515	0.010	
2	50.00	0.495	0.495	0.004	
3	50.00	0.497	0.497	0.001	
4	60.00	0.496	0.467	0.030	
5	46.00	0.261	0.246	0.010	0.457
6	33.00	0.152	0.125	0.005	0.700
7	2000.00				1.000

The simulation of the XRump code yields a film thickness of 357 nm (graded) and it is in agreement with values obtained from the surface profilometer (356.6 nm). The presence of Si at depths within the ZnO:Eu^3+^ film is attributed to diffusion (Thermal Diffusivity 0.9 cm^2^/sec) and atomic intermixing at the interface. The total average Eu composition is ∼1% with majority being found at a depth of 278 nm (upto layer 4).

### X-Ray Diffraction Analysis

**Figure 3:**
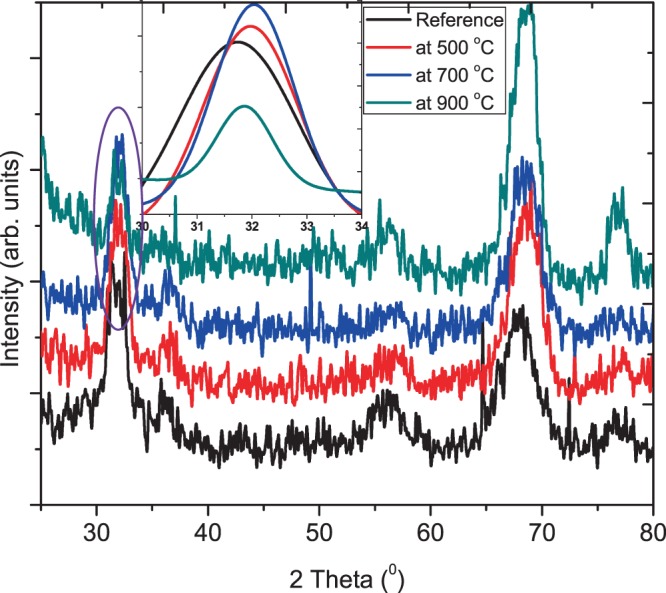
Room temperature XRD pattern of pristine and annealed ZnO:Eu^3+^ thin films. Inset is a fitting of the (100) peak.

The room temperature XRD pattern of ZnO:Eu^3+^ thin film annealed at various temperatures are depicted in Fig. 3. It is evident that the ZnO:Eu^3+^ film has a predominantly hexagonal wurtzite structure (JCPDS database No: 00–003–0888). For different annealing temperatures, the detected diffraction angles (2θ), FWHM of the (100) peak, d values, grain sizes, dislocation densities and tensile strains in the films are tabulated in Table 2.

**Table 2:** Summary of structural values of ZnO:Eu^3+^ thin film; as-grown and annealed at different temperatures (500–900 °C).

Annealing Temperature (°C)	Crystallite size, *D*_*sh*_/(Å)					
	(001) peak	FWHM peak	d-spacing *a*/(Å)	Crystallite size *D* (Å)	*δ* (10^16^)	ε (10^−3^)
Reference	31.7	2.16	2.82	38.4	6.78	9.03
500	32.0	1.51	2.79	54.8	3.32	6.32
700	33.2	1.45	2.68	57.1	3.07	6.08
900	31.9	1.83	2.83	58.2	4.90	7.68

Additionally, it is observed that there are no diffraction peaks associated with europium oxides or other impurities in the XRD pattern of Fig. 3. This is an indication that the Eu^3+^ ions are probably incorporated into the ZnO lattice. All peaks in the XRD pattern are of ZnO and could be readily indexed as the hexagonal wurtzite ZnO phase with lattice constants (a) 3.24890 Å and (c) 5.20620 Å, which are in good agreement with the reported data in the literature^16–18^. Usually the diffraction peaks of Eu^3+^ doped ZnO samples may have a lower angle (2θ) compared to undoped ZnO thin film, in our case a shift of about 0.5 (2θ) as a result of the larger ionic radius of Eu^3+^ (0.95 Å) in comparison to that of Zn^2+^ (0.74 Å)^19^ is observed.

Upon annealing of ZnO:Eu^3+^ thin films, the prominent (100) peak is gradually shifted to higher 2θ (31.74° to 32.04°) while its FWHM gradually decreases from 2.82 to 1.45 nm for (500–700 °C) temperature range, an indication of enhanced crystallinity with annealing.

The residual strain decreased from 9.03 × 10^−3^ to 6.08 × 10^−3^ with increasing temperature up to 700 °C. Above 900 °C the strain increases to 7.68 × 10^−3^. Without annealing, surface atoms have less energy hence low surface mobility, resulting in generation of defects in ZnO films. On annealing at 500–700 °C, the atoms gain sufficient mobility to engender atomic rearrangement during crystallization of ZnO. The effect is further supported by reduction in the FWHM. Further increase in annealing temperature to 900 °C resulted in breaking of partially coordinated Zn-O bonds on the surface rather than enabling the atoms to move freely to their corresponding stable sites, producing defects and stress in ZnO film hence the increase in strain and dislocation density^20^.

### Optical spectroscopy

**Figure 4:**
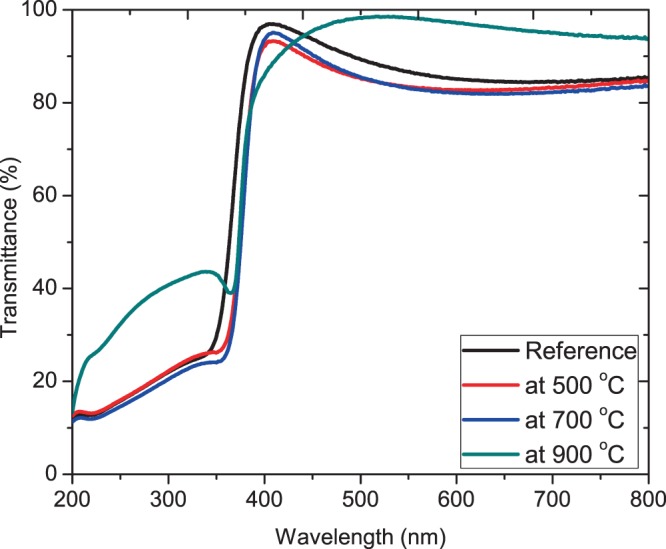
Optical transmittance of pristine and ZnO:Eu^3+^ annealed at 500–900 °C.

Figure 4 represents the transmittance spectra in (%) of the as deposited and annealed ZnO:Eu^3+^ thin films on quartz substrates capable of withstanding the high annealing temperatures without softening. This was done as a function of wavelength. The rapid cutting in intensity near 372 nm is attributed to the band edge absorption. The increase in transmittance at λ < 330 nm can be attributed to structural changes in glass at high temperature (900 °C).

**Figure 5:**
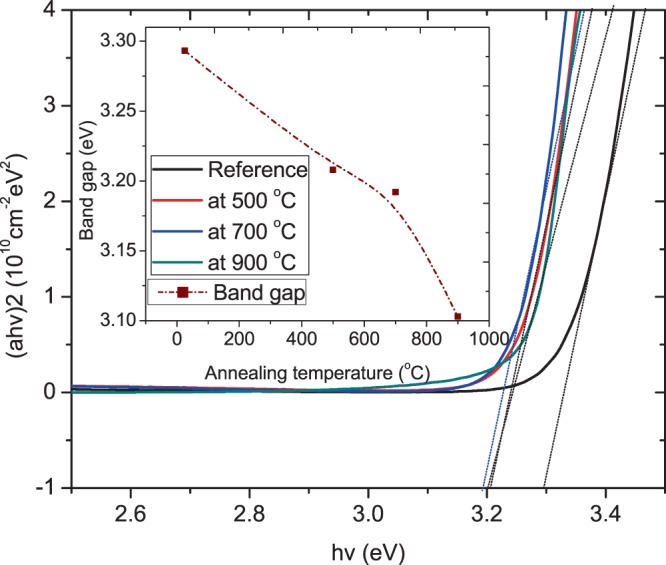
Plot of (αhν)^2^ versus hν for the pristine ZnO:Eu^3+^ thin film and after annealing at 500–900 °C. The inset shows the variation of optical band gap as a function of annealing temperature.

From Fig. 4, high transparency in the visible wavelength range was observed in all the thin films with a sharp ultraviolet absorption edge in the UV region approximately at 372 nm. This abrupt absorption edge coupled with excellent optical transmission within the visible range of the spectrum is an indication of the films of high quality. The interferential behavior of spectra is attributed to light reflectance occurring between ZnO:Eu^3+^ - substrate interface and ZnO:Eu^3+^ - air surface^21^. High transmittance of about 95% recorded is crucial to effective application into solar cells where highly transparent and conductive is major requirement of the electrodes in device application. It can be seen that as the annealing temperature increases, the absorption edge shift to lower energy. After doping with Eu^3+^ ions, a systematic low-energy shift occurs in the band gap, indicating that the optical band gap is narrowed^22^. As shown in Fig. 5, the UV-Vis transmission spectrum was used to obtain the change in optical band gap with annealing temperature. Then, we use the direct allowable electron transition between the highest occupied state of the valence band and the lowest unoccupied state of the conduction band when a photon of energy, *hν*, falls on the film. The absorption coefficient, α, is related to the optical bandgap energy, Eg, by Tauc equation:

$$\alpha hv={C}_{1}{(hv-{E}_{g})}^{m}$$

where *α* is the absorption coefficient, *hv* is the incident photon energy, *C*_1_ is a constant, *E*_*g*_ is the optical gap energy, and the exponent *m* is 1/2 for allowed transition enabling absorption based on the Fermi Golden rules.

When the films are annealed from 500–700 °C, the optical bandgap is observed to decrease from 3.29 to 3.19 eV before a larger decrease to 3.103 eV upon annealing at 900 °C as shown in Fig. 5. The decrease in Eg is a show of enhanced films quality as the structural defects are annihilated^23^ such as decrease of the O vacancies^24^. This is in agreement with the experimental results of XRD analysis. The tailoring of the bandgap of semiconductors could also be attributed to the introduction of new energy levels in the band gap near conduction edge such as shallow level donor impurities^25^.

### Photoluminescence properties

**Figure 6:**
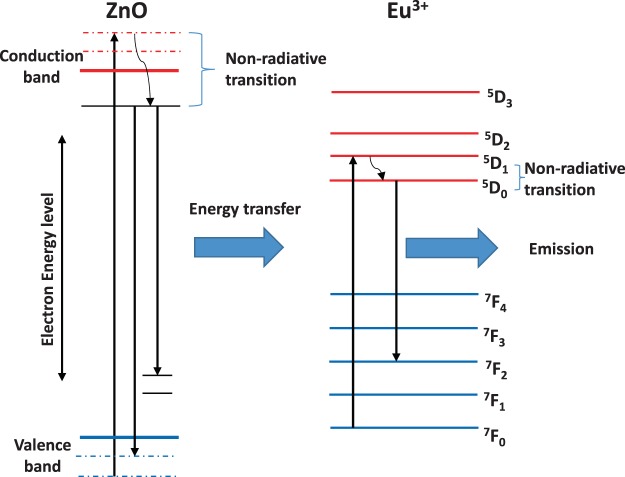
Schematic illustration of the proposed mechanism of energy transfer from the ZnO host to the Eu^3+^ ions^26^.

**Figure 7:**
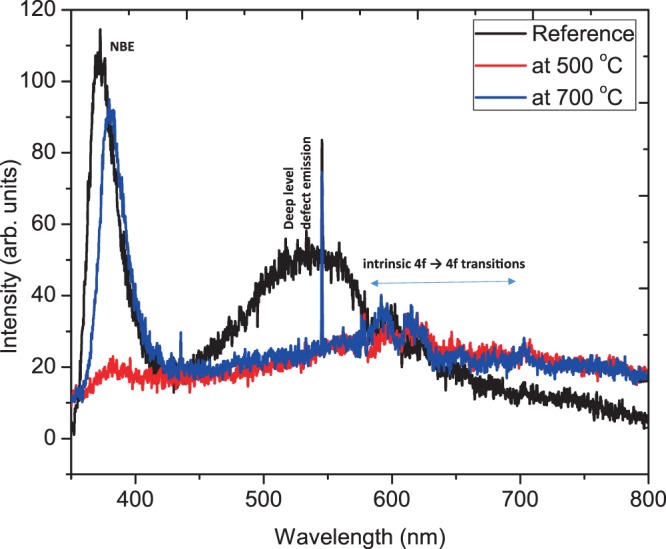
Room temperature PL spectrum of ZnO:Eu^3+^ annealed at 500–700 °C measured at 244 nm excitation wavelength.

The Changes in the nanoscale energy level of the thin film structure may seriously affect the light emitting performance. The relationship between the structure and emission behavior of ZnO:Eu^3+^ thin films and the annealing temperature was investigated using PL. Figure 7 shows the photoluminescence spectrum of Eu-doped ZnO thin film in the range of 350–800 nm, depicting several energy bands relating to the transitions from excited ^5^D_0_ level to the ^7^F_J_ (J = 0, 1, 2, 3, 4) levels of the Eu^3+^ 4f^6^ configuration^26^. It is mainly shown that the spectrum of the ^5^D_0_–7F_2_ transition is modified when the annealing temperature is increased, which indicates that there is a structural rearrangement around the Eu^3+^ centers. This is consistent with the proposed conversion shown in Fig. 6.

**Figure 8:**
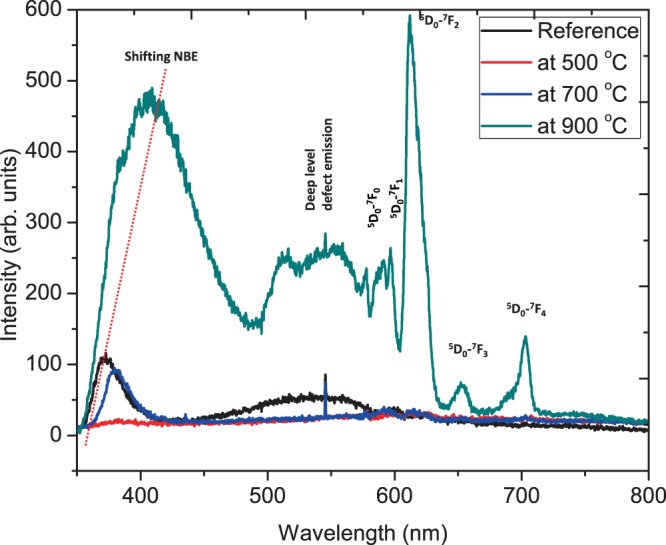
Room temperature PL spectrum of ZnO:Eu^3+^ annealed at 500–700 °C measured at 244 nm excitation wavelength.

From Figs. 6–7, the UV emission attributed to near-band-edge (NBE) exciton recombination and a broad deep level emission is depicted as discussed in our previous publication^3^. Figure 7 shows the PL spectra of films annealed at between 500–700 °C while Fig. 8 includes films annealed at 900 °C.

It can be seen from Fig. 7 that at 372 nm and 536 nm, the intensity of near-band edge emission and deep-level defect emission suddenly decreased, and then began to increase after 700 °C, while the emission of the ^5^D_0_–7F_2_ transition at 613 nm increasing is a confirmation of energy transfer from ZnO to Eu^3+^ ions^27–29^.

It can be seen from Fig. 8 that when annealing at 900 °C, sharp peaks are observed at the wavelengths of 576 nm, 594 nm, 613 nm, 651 nm, and 703 nm, which correspond to the inherent 4 f → 4 f transition of Eu^3 +^ in the ZnO host. Eu^3+^ ‘s intrinsic 4 f → 4 f transitions are assigned to ^5^D_0_ - ^7^F_0_, ^5^D_0_ - ^7^F_1_, ^5^D_0_ - ^7^F_2_, ^5^D_0_ - ^7^F_3_, and ^5^D_0_ - ^7^F_4_ respectively^19,30,31^. The strong peak at 613 nm indicates the occupancy of Eu^3+^ ions at the antisymmetric site of the host ZnO^19^. According to Judd-Ofelt theory, if Eu^3+^ ions occupy the center of symmetry of the ZnO lattice, magnetic dipole transitions are allowed. In this case, and in accordance with the Laporte selection rule in 4f^6^ transition, the electric dipole transitions is prohibited. However, electric dipole transitions are allowed only when Eu^3+^ ions occupy a position without an inversion center and their strength is significantly affected by the symmetry in the local environment^32^. If Eu^3+^ ions occupy an inverse symmetric site in the lattice, the magnetic transition ^5^D_0_ - ^7^F_1_ (about 594 nm) will be the main transition. Therefore, the dominance of ^5^D_0_ - ^7^F_2_ transitions in the spectrum indicates the possibility of electrical dipole transitions. The ratio of intensity (^5^D_0_- ^7^F_2_)/(^5^D_0_ - ^7^F_1_) can be used to establish a measure of deformation based on the antisymmetric nature of the Eu^3+^ ion local environment. It is known that the lower symmetry around Eu^3+^ ions will produce a larger intensity ratio, which is called asymmetry factor or asymmetry ratio^33^. Due to the ^5^D_0_-^7^F_2_ transition at 900 °C, the red peak at 613 nm has a very high intensity, which confirms that the Eu^3+^ emission is parity and only deforms in the lattice environment and has no inversion symmetry^32^. Since this is a forced electric dipole transition, this transition is very sensitive to the annealing temperature. It is known that Eu^3+^ ions may occupy two positions, namely the internal and surface lattice positions of ZnO materials^29,34,35^. In theory, Eu^3+^ ions are doped into the internal lattice sites of ZnO, or located at the Zn substitution sites with C3v symmetry. The transition of J = 0 and J = 1 will be divided into two crystals-field energy levels (J = 0 → J = 0, J = 0 → J = 1), but as can be seen from Fig. 6, there are five peaks at ^5^D_0_-^7^F_j_, which indicates that Eu^3+^ ions have been incorporated into a lower symmetry site than C3v^36^.

### Application of ZnO:Eu^3+^ into Dye sensitized solar cells

**Figure 9:**
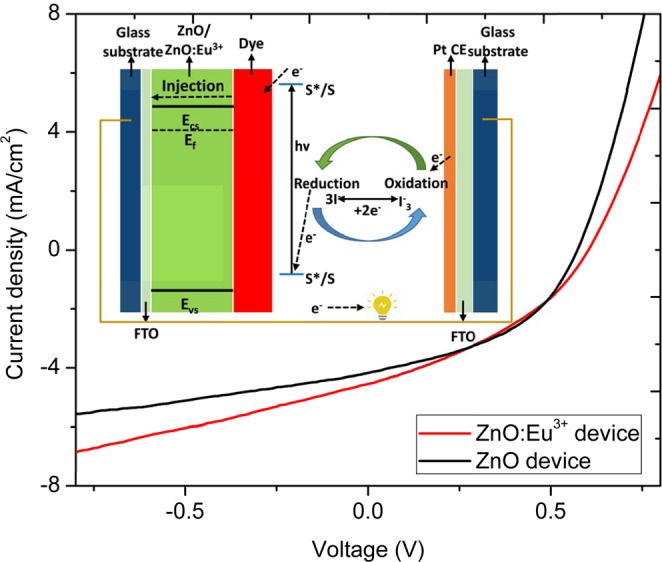
*J-V* characteristics curves of pristine and Eu^3+^ doped ZnO used in dye sensitized solar as a photoanode. In set: Structure and operating principle of a typical DSSC adapted from ref. ^41^.

Figure 9 shows *J-V* characteristics of dye sensitized solar cell fabricated and tested in air ambient as a proof of concept. The sputtered films on cleaned FTO were used as an electrode. ZnO and Eu doped ZnO was deposited on it and the films were annealed at 500 °C as high temperature would lead to softening of glass supporting FTO. The aim of the heat treatment was to get improved adsorption between the film and dye molecules. The thickness and the active area of the film used were approximated to 357 nm and 0.25 cm^2^ respectively.

The DSSC was made of a salt electrolyte sandwiched between a dye-sensitized a counter electrode (CE) and photoanode (working electrode)^37^. The Photoanode is composed of a layer of wide-band gap semiconductor that is mesoporous attached to the conducting glass. Although we used ZnO:Eu3^+^ as mesoporous semiconductor, other oxides may also be used such as TiO_2_^38–40^. A thin layer of charge transfer dye was then adsorbed on the surface of the mesoporous ZnO:Eu3^+^ semiconductor. This photoanode section was then attached to the redox electrolyte or hole conductor. Eventually the device was completed by connecting the sensitized photoanode with the counter electrode (cathode) as shown in Fig. 9 (inset).

The DSSC with ZnO:Eu^3+^ yielded a better performance with PCE of 1.33% (Jsc = 4.56 mA cm^−2^, V_oc_ = 0.59 V and FF = 49.3%), compared to the DSSC with pristine ZnO that produced a PCE of 1.19% (Jsc = 4.14 mA cm^−2^, Voc = 0.57 V, FF = 50.6%) indicating 11.1% efficiency enhancement. The enhancement in efficiency is attributed to spectral conversion by the Eu doped ZnO. The device performance values obtained were relatively low. This low performance could be attributed to the exposure of the materials to air during fabrication and testing process. This exposure was deliberate as a proof of concept that the device fabrication and testing carried out in air is indeed feasible.

## Conclusion

We successfully report deposition of polycrystalline ZnO:Eu^3+^ thin films of high orientation on (001) Si, quartz and FTO coated glass substrate using RF magnetron sputtering technique. A systematic analysis of the film crystallization dynamics is carried out at elevated temperature annealing in Argon filled furnace. The deposited thin films showed good transparency that became even better with annealing making them suitable for solar cell applications. Scanning electron microscope (SEM), grazing incidence X-ray diffraction (GXRD), and the Photoluminescence (PL) were used to characterize the effect of the post-deposition annealing treatment on the structural properties of the ZnO:Eu^3+^ thin films. The results shows that a small amount of Eu has been successfully incorporated in the ZnO host matrix. Structural studies reveal that films deposited by sputtering have a better structural quality that is even enhanced with annealing the films. The thin films indicated an increasing grain size and roughness of ZnO:Eu^3+^ with annealing temperature between 500–900 °C as well as a shifted diffraction peak position. Finally, we incorporated the thin films into a dye sensitized solar cell fabrication as a proof of concept. The fabricated devices with ZnO:Eu^3+^ thin films showed a remarkable performance with better transparency and efficiency enhancement that we highly attributed to spectral conversion when incorporated as a bi-functional layer.

## Supplementary information

Supplementary information.
