# Supplementary material for: Annealing effect on the structural and optical behavior of ZnO:Eu3+ thin film grown using RF magnetron sputtering technique and application to dye sensitized solar cells
Source: Sci Rep. 2020 May 22;10:8557. doi: 10.1038/s41598-020-65231-6 (PMC7244725; doi:10.1038/s41598-020-65231-6)
Supplement: Supplementary file 1 — Supplementary information. [file 41598_2020_65231_MOESM1_ESM.docx]

**Annealing effect on the structural and optical behavior of ZnO:Eu^3+^ thin film grown using RF magnetron sputtering technique and application to dye sensitized solar cells.**

**Francis Otieno*^1,2^, Mildred Airo^2^, Rudolph M. Erasmus^1^ , Alexander Quandt^1,2^, David G. Billing^2^ and Daniel Wamwangi^1,2^**

^1^Materials for Energy Research group, Material Physics Research Institute, School of Physics, University of the Witwatersrand, Private Bag 3, Wits, 2050, Johannesburg, South Africa.

^2^School of Chemistry, University of the Witwatersrand, Private Bag 3, Wits, 2050, South Africa.

* corresponding author (frankotienoo@gmail.com)

Figure S1: Taping mode atomic force Microscopy micrographs of Eu doped ZnO films annealed at temperature range of 500-900 ˚C


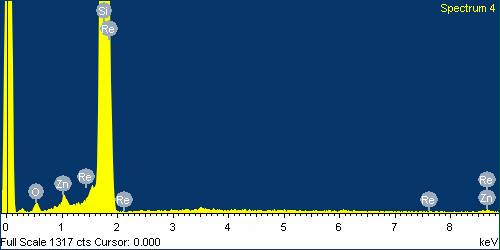


Figure S2: Energy-dispersive X-ray spectroscopy of Eu doped ZnO films annealed at temperature range of 500 ˚C
